# Imaging activity in astrocytes and neurons with genetically encoded calcium indicators following *in utero* electroporation

**DOI:** 10.3389/fnmol.2015.00010

**Published:** 2015-04-15

**Authors:** J. Michael Gee, Meredith B. Gibbons, Marsa Taheri, Sierra Palumbos, S. Craig Morris, Roy M. Smeal, Katherine F. Flynn, Michael N. Economo, Christian G. Cizek, Mario R. Capecchi, Petr Tvrdik, Karen S. Wilcox, John A. White

**Affiliations:** ^1^Neuronal Dynamics Laboratory, Department of Bioengineering, University of UtahSalt Lake City, UT, USA; ^2^MD-PhD Program, University of UtahSalt Lake City, UT, USA; ^3^Glial-Neuronal Interactions in Epilepsy Laboratory, Department of Pharmacology and Toxicology, University of UtahSalt Lake City, UT, USA; ^4^Interdepartmental Program in Neuroscience, University of UtahSalt Lake City, UT, USA; ^5^Mario Capecchi Laboratory, Department of Human Genetics, University of UtahSalt Lake City, UT, USA; ^6^Department of Human Genetics, Howard Hughes Medical Institute, University of UtahSalt Lake City, UT, USA

**Keywords:** GCaMP6, rat model, GCaMP3, calcium imaging, astroglia, neural network, gene expression, tdTomato

## Abstract

Complex interactions between networks of astrocytes and neurons are beginning to be appreciated, but remain poorly understood. Transgenic mice expressing fluorescent protein reporters of cellular activity, such as the GCaMP family of genetically encoded calcium indicators (GECIs), have been used to explore network behavior. However, in some cases, it may be desirable to use long-established rat models that closely mimic particular aspects of human conditions such as Parkinson's disease and the development of epilepsy following status epilepticus. Methods for expressing reporter proteins in the rat brain are relatively limited. Transgenic rat technologies exist but are fairly immature. Viral-mediated expression is robust but unstable, requires invasive injections, and only works well for fairly small genes (<5 kb). *In utero* electroporation (*IUE*) offers a valuable alternative. *IUE* is a proven method for transfecting populations of astrocytes and neurons in the rat brain without the strict limitations on transgene size. We built a toolset of *IUE* plasmids carrying GCaMP variants 3, 6s, or 6f driven by CAG and targeted to the cytosol or the plasma membrane. Because low baseline fluorescence of GCaMP can hinder identification of transfected cells, we included the option of co-expressing a cytosolic tdTomato protein. A binary system consisting of a plasmid carrying a *piggyBac* inverted terminal repeat (ITR)-flanked CAG-GCaMP-IRES-tdTomato cassette and a separate plasmid encoding for expression of *piggyBac* transposase was employed to stably express GCaMP and tdTomato. The plasmids were co-electroporated on embryonic days 13.5–14.5 and astrocytic and neuronal activity was subsequently imaged in acute or cultured brain slices prepared from the cortex or hippocampus. Large spontaneous transients were detected in slices obtained from rats of varying ages up to 127 days. In this report, we demonstrate the utility of this toolset for interrogating astrocytic and neuronal activity in the rat brain.

## Introduction

The brain comprises networks of glia and neurons. Investigation of glial and neuronal network dynamics is necessary for understanding complex behavior and disease. The rat brain is particularly well-suited for modeling neurophysiology and neuropathology. The predominant use of rats throughout the history of neuroscience research has already created a wealth of knowledge about many behaviors and disorders including the kainic acid model of status epilepticus and the 6-OHDA model of Parkinson's disease (Schultz, 1982; Jacob, 1999; Cenci et al., 2002; Williams et al., 2007). Unfortunately, rat models are restricted by limited methods for interrogating the activity of numerous cells simultaneously, especially glia. Traditional electrophysiological techniques for recording neuronal activity are inadequate for less electrically active glial cells. However, glial cells, as well as neurons, exhibit substantial intracellular calcium concentration transients which can be measured as a proxy for cellular activity. Fluorescent synthetic dyes, derived from the EGTA homolog BAPTA, such as Fluo-4, Oregon Green BAPTA, and Indo-1 are frequently used for calcium imaging (Tsien, 1980; Uematsu et al., 1991; Kreitzer et al., 2000; McDonough et al., 2000; Stosiek et al., 2003; Kuga et al., 2011; Lillis et al., 2012), but have several drawbacks. Synthetic dye loading techniques are particularly difficult to employ in mature or pathological tissue, limiting their use to a subset of experimental preparations—those in healthy young tissue (Peterlin et al., 2000; Reeves et al., 2011). Additionally, synthetic dyes cannot be targeted to specific cell types or intracellular regions (Mank and Griesbeck, 2008; Mao et al., 2008) except in special cases (Ding, 2012). Over the past decade, genetic approaches have evolved to ameliorate these issues.

Genetically encoded calcium indicators (GECIs), such as the GFP-based GCaMP family, are a powerful tool for investigation of cellular activity (Nakai et al., 2001; Tian et al., 2009; Chen et al., 2013). Viral vectors are often used to deliver GCaMP to target tissues, but there exist several reasons why use of this approach may not be desirable. Viruses require invasive injections, often times near the location and time of imaging. Adeno-associated virus, while extremely useful for transgene delivery and relatively non-immunogenic, is hampered by a low carrying capacity of less than 5 kb (Grieger and Samulski, 2005). Alternatively, rat transgenic tools are quickly improving but are limited in number and require significant time and labor to develop. *In utero* electroporation (*IUE*) offers a valuable substitute for both of these approaches (Saito and Nakatsuji, 2001; Tabata and Nakajima, 2001; Bai et al., 2003; Borrell et al., 2005; Nakahira and Yuasa, 2005). After injecting DNA into the lateral ventricles of the embryonic brain, an electric field is applied across the uterine walls to facilitate transfection of glia and neuron progenitors which eventually divide and migrate throughout the brain. Unfortunately, transfected plasmid DNA tends to remain episomal and becomes diluted in persistently dividing cells, such as astrocytes, the predominant glial cell. To circumvent this problem, several different transposon systems, including *Tol2* from the Japanese rice fish *Oryzias latipes* or *piggyBac* from the cabbage looper moth *Trichoplusia ni*, can be applied to mediate stable genomic integration of inverted terminal repeat (ITR)-flanked DNA (Cary et al., 1989; Fraser et al., 1995, 1996; Ding et al., 2005; Yoshida et al., 2010; Chen and LoTurco, 2012). *piggyBac* can transpose transgenes of up to 100 kb between plasmid DNA and genomic DNA and has been adapted for use in mammalian systems (Lorenzen et al., 2003; Ding et al., 2005; Wu et al., 2007; VandenDriessche et al., 2009; Li et al., 2011b). When combined with *IUE, piggyBac*-mediated transposition results in propagation of the transgene to the entire cell lineage of transfected progenitors (Wu et al., 2007; Chen and LoTurco, 2012).

In this report, we describe a toolset of plasmid constructs, carrying cytosol- or plasma membrane-targeted ITR-flanked GCaMP3 or versions of GCaMP6, designed specifically for calcium imaging of the rat brain following *IUE*. We demonstrate that these constructs are stably expressed well into adulthood and are functional in astrocytes and neurons in acute cortical slices or hippocampal organotypic slice cultures (OSCs). We present data collected with the *piggyBac* ITR-flanked CAG-Lck-GCaMP6f (pPBC-LG6f) plasmid as an example of the utility of these tools. This toolset offers an attractive approach for future studies investigating astrocytic-neuronal network behavior in various rat brain preparations.

## Materials and methods

### Animals

Pregnant Sprague Dawley CD dams were obtained from Charles River Laboratories, Inc. (Wilmington, MA) and maintained at the University of Utah animal facility. Both male and female animals were used. All experimental protocols were approved by the University of Utah Institutional Animal Care and Use Committee (IACUC).

### Plasmid generation

**Figure 1:**
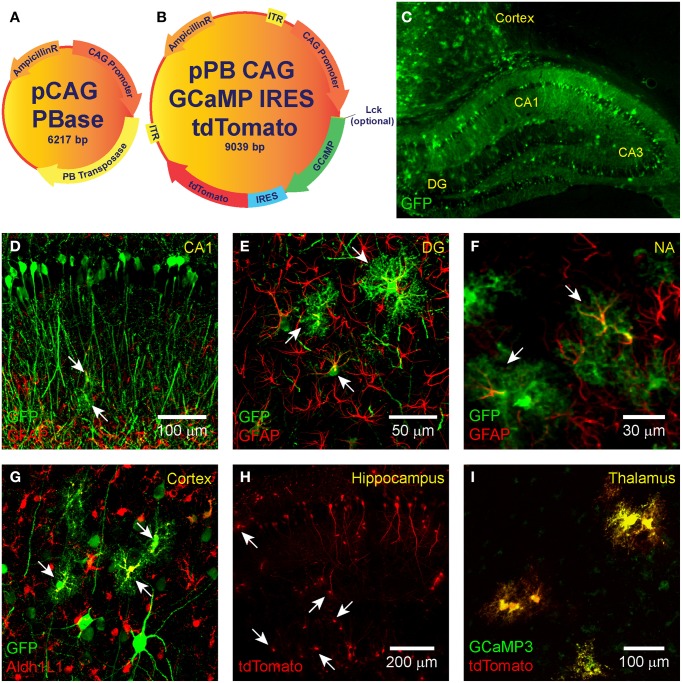
***IUE*-mediated transgene delivery to astrocytes and neurons in various regions of the brain**. The binary co-transfection system consists of a **(A)** helper plasmid carrying the *piggyBac* transposase enzyme and a **(B)** donor plasmid carrying the GCaMP (3, 6s, or 6f)-IRES-tdTomato construct flanked with *piggyBac* inverted terminal repeats. A subset of donor plasmids include an N-terminal Lck-tagged GCaMP for plasma membrane targeting. All transgenes are expressed under the control of CAG. **(C–G)** *IUE* of CAG-GFP-IRES-Neo transfected astrocytes and neurons in the **(C,D)** hippocampus, **(C,G)** cortex, the **(E)** dentate gyrus, and **(F)** nucleus accumbens. Fluorophore-conjugated anti-GFP antibody enhanced GFP is shown in green. In **(D–F)** astrocyte-labeling anti-GFAP antibody is shown in red and overlap (transfected astrocytes) in yellow. **(G)** GFAP overlap with GFP was sometimes difficult to detect, so we also stained astrocyte-specific Aldh1L1 which is shown in red with transfected astrocytes in yellow. **(H)** Hippocampal expression pattern of tdTomato expression (naïve fluorescence) and **(I)** co-expression (yellow) of GCaMP3 (green) and tdTomato (red) in putative thalamic neurons following *IUE* transfection of CAG-GCaMP3-IRES-tdTomato (naïve fluorescence). White arrows denote putative astrocytes.

The GECIs GCaMP3 or GCaMP6 (Tian et al., 2009; Chen et al., 2013) and the red fluorescent reporter protein tdTomato (Shaner et al., 2004) were expressed from circular plasmid DNA that was prepared by standard DNA cloning methods. A binary plasmid mix was used in a 3:1 ratio. The donor plasmid harbors GCaMP and tdTomato connected via an internal ribosomal entry site (IRES; Figure 1B) and is driven by the strong ubiquitous promoter cytomegalovirus early enhancer/chicken beta actin (CAG; Niwa et al., 1991). IRES is a bicistronic sequence that allows for simultaneous expression of two proteins separately but from the same RNA transcript. The CAG-GCaMP-IRES-tdTomato construct was flanked with *piggyBac* ITRs obtained from the pZG-s plasmid (Figure 1B; Wu et al., 2007). To target GCaMP to the plasma membrane, we spliced the Lck N-terminal sequence to the N-terminus of GCaMP (Figure 1A; Shigetomi et al., 2010b). The helper plasmid (pPBase) encodes for the *piggyBac* transposase enzyme (Figure 1A; Wu et al., 2007) and is likewise driven by CAG. For some experiments we used the pPB-CAG-GFP-IRES-Neo plasmid (pPBC-GFP; a gift from Sen Wu in the Capecchi lab).

### Immunohistochemistry and confocal imaging

For confirmation of targeting experiments (Figures 1C–I), seven rats were electroporated with pPBC-GFP. Postnatal day (P) 20 rats were anesthetized with Nembutal (50 mg/kg i.p.; Ovation Pharm, Deerfield, IL, USA) and transcardially perfused with 1 × PBS followed by 4% paraformaldehyde (PFA) diluted with 1 × PBS (pH 7.6). Brains were removed and post-fixed for 24 h in 4% PFA at 4°C. Brain sections (40 μm) were then prepared on a vibratome and mounted on glass slides. Slices were blocked with BSA (A2153 Sigma-Aldrich, St. Louis, MO, USA) for 1 h and then incubated with either the primary antibody chicken anti-GFP IgY (1:500 overnight; A10262 Life Technologies, Carlsbad, CA, USA) and secondary antibody DyLight 488-conjugated AffiniPure donkey anti-chicken IgY (1:750 2 h) for GFP-staining or with the Cy3-conjugated mouse anti-glial fibrillary acidic protein (GFAP; 1:1000 overnight; C9205 Sigma-Aldrich, St. Louis, MO, USA) for GFAP-staining. Aldh1L1 was labeled by rabbit anti-Aldh1L1 (ab87117, Abcam, Cambridge, MA, USA). Following antibody labeling, slides were incubated in 1 × DAPI for 30 min and mounted with ProLong Gold Antifade Mountant (P36930 Invitrogen, Carlsbad, CA, USA).

**Figure 2:**
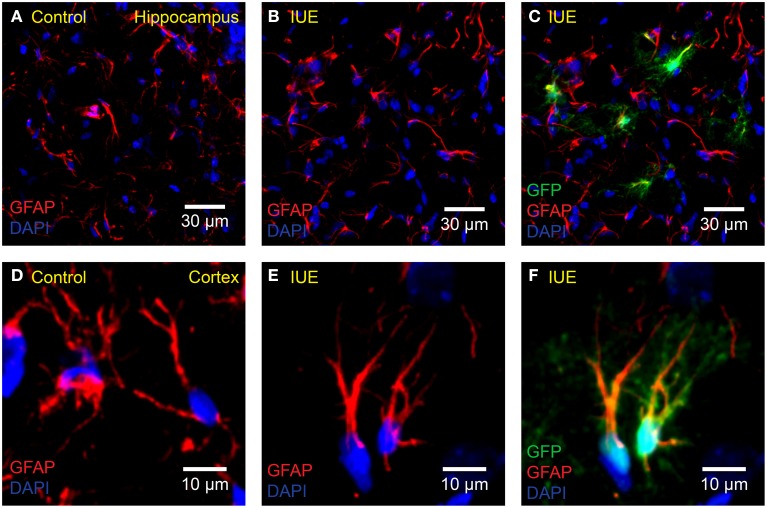
**No apparent astrogliosis following *IUE***. Confocal imaging of astrocyte GFAP in the **(A–C)** hippocampus or **(D–F)** cortex of **(A,D)** unelectroporated controls or **(B,C,E,F)** following *IUE* of CAG-GFP-IRES-Neo (green). For better comparison with **(A)**, **(B)** displays only the red (anti-GFAP) and blue (DAPI) channels of **(C)**. Likewise, for comparison with **(D)**, **(E)** displays the anti-GFAP and DAPI channels of **(F)**. Transfected astrocytes are partially yellow/orange. There were no striking qualitative differences in GFAP expression and morphology between age-matched (P18) control and *IUE* groups, even in transfected astrocytes.

To test for a possible immune response following *IUE* (Figure 2), four rats electroporated with pPBC-GFP and three unmanipulated age-matched controls were sacrificed at P18. The brains were removed and post-fixed for 24 h in 4% PFA solution at 4°C. They were then transferred to a sucrose solution gradient (15 and 30% for 24 h each) at 4°C or until brains sank in solution. Brains were embedded with OCT compound in a “brain box,” then frozen and stored at −20°C. Coronal sections (20 μm) were prepared using a cryostat and mounted onto charged glass slides (Superfrost Plus Micro Slide, VWR, Batavia, IL, USA). The sections were permeabilized at room temperature for 40 min with 0.1% Triton-X diluted in 1 × PBS. For GFAP labeling, the same CY3-conjugated anti-GFAP antibody described above was diluted in CytoQ (1:800) and applied for 1.5 h at room temperature. For Iba-1 labeling, primary goat polyclonal anti-Iba1 antibody diluted in CytoQ (1:600, ab5076, Abcam, Cambridge, MA, USA) was applied for 24 h at 4°C, followed by secondary rabbit anti-goat Alexa Fluor 555 antibody (1:500, ab150146, Abcam, Cambridge, MA, USA) for 2 h at room temperature. Finally, ProLong Gold Antifade Mountant with DAPI was applied to the sections as the mounting medium. All imaging was performed using an Olympus Fluoview FV1000 confocal microscope with either 20 × 0.75 NA or 40 × 0.80 NA water immersion objectives (Olympus, Tokyo, Japan). Images were processed using ImageJ.

### *In utero* electroporation

Transfection of the embryonic rat brain was accomplished via an established *IUE* protocol (Bai et al., 2003). After induction of a deep anesthetic state with ketamine/xylazine (70/30 mixture, 0.1 mg/g body weight, i.p.), the abdomen of a multiparous pregnant rat was shaved and swabbed with betadine. A laparotomy was performed and embryos (gestational day 13.5–14.5) within the uterine horn were exposed and gently placed on a sterile and irrigated gauze pad. Because the embryos at this age stretch the uterus, they can be clearly visualized with only the aid of a bright fiber optic lamp. A glass capillary tube was pulled (Sutter Instrument, Novato, CA, USA) to a fine point by high heat and filled with a mixture of fast green (1%; Sigma-Aldrich, St. Louis, MO, USA) and DNA plasmid solution (1 μg/μl final). The pipette tip was inserted through the uterus into one of the lateral ventricles of the embryo brain and a small volume (1–3 μl) of the mixture was pressure injected by a picospritzer (Picospritzer III; Parker, Mayfield Heights, OH, USA) or by applying positive pressure through a mouth pipette. Successful injection was confirmed visually by observing fast green filling of the ventricle. For electroporation, a pair of sterilized gold copper alloy plate electrodes (1 × 0.5 cm) were positioned across the head of the embryo on the uterus exterior, and a very brief (1 ms) voltage pulse (65 V) was discharged across the electrodes. In order to specifically target the developing hippocampus and cortex, the paddles were rotated across the top of the head with the positive electrode placed opposite the injected side, as previously described (Navarro-Quiroga et al., 2007; Pacary et al., 2012). The embryos within the uterus were returned to the body cavity and the incision closed with sterile Ethicon silk suture 4.0 (Ethicon, Somerville, NJ, USA). Animals were weaned from the mother on P21 and males and females housed separately until used for experiments.

### Acute brain slice preparation

Brain slices were prepared using established techniques (Netoff et al., 2005; Kispersky et al., 2012; Smeal et al., 2012). Briefly, electroporated rats of either sex were deeply anesthetized using isoflurane and decapitated at various time points spanning P34 through P127. Brains were rapidly dissected and placed in ice-cold (4°C) oxygenated sucrose Ringer's solution containing (in mM): 200 sucrose, 26 NaHCO_3_, 10 D-glucose, 3 KCl, 3 MgCl, 1.4 NaH_2_PO_4_, and 1 CaCl_2_. The pH was maintained at 7.4 by saturation with 95% O_2_–5% CO_2_. A vibrating microtome, (VT1200; Leica, Wetzlar, Germany) with the bath chilled to 1.5°C (Minichiller; Huber, Offenburg, Germany), was used to prepare 400 μm thick coronal slices in oxygenated sucrose Ringer's solution (4°C). Following sectioning, slices were transferred to a holding chamber filled with oxygenated artificial cerebrospinal fluid (ACSF; room temperature) containing (in mM): 126 NaCl, 26 NaHCO_3_, 10 D-glucose, 3 KCl, 2 MgCl, 2 CaCl_2_, and 1.4 NaH_2_PO_4_ for at least 1 h. During imaging, slices were placed in a heated (32°C) immersion style recording chamber (Warner Instruments, Hamden, CT, USA) mounted on a microscope stage and perfused with ACSF.

### Organotypic slice cultures

Hippocampal OSCs were prepared from P10 rats as previously described (Alex et al., 2011). Slices (400 μm) were maintained on membrane inserts (Millicell CM, Millipore, Bedford, MA, USA) in a 6 well plate (4 slices per insert/well), containing 1 ml of culture medium, until imaging experiments were conducted 7–10 days later. Slices were imaged in oxygenated ACSF containing (in mM): 126 NaCl, 26 NaHCO_3_, 10 D-glucose, 3 KCl, 2 CaCl_2_, 2 MgCl, and 1.4 NaH_2_PO_4_.

### Two-photon imaging

Two-photon imaging was performed using a custom-built two-photon microscope (Lillis et al., 2008; Smeal et al., 2012) assembled around a mode-locked Ti:Sapphire laser source emitting 140 fs pulses at an 80 MHz repetition rate with a wavelength adjustable from 690 to 1040 nm (Chameleon Ultra I; Coherent, Santa Clara, CA, USA). Laser emission wavelengths of 940–950 nm were used to excite GCaMP (Tian et al., 2009; Chen et al., 2013) or 1040 nm to excite tdTomato (Drobizhev et al., 2009, 2011). In this setup, laser power is attenuated using an electro-optic modulator (ConOptics, Danbury, CT, USA) and scanning is accomplished using x-y galvanometer-mounted mirrors (GSI Lumonics, Billerica, MA, USA) controlled by custom LabVIEW software (Lillis et al., 2008) and a PCI-6221 data acquisition board (National Instruments, Austin, TX, USA). Full field of view images were acquired with an x-y raster scan. We used 20 × 0.95 NA or 60 × 0.9 NA objectives (Olympus, Tokyo, Japan). Emitted photons were bandpass filtered (Semrock, Rochester, NY, USA) at (peak/bandwidth): 525/50 nm (GCaMP), 593/46 nm (tdTomato) and collected by a wide band (300-650 nm) and low noise photomultiplier tube (H7360-01; Hamamatsu, Hamamatsu City, Japan). To avoid the “dead zone” on the surface of brain slices, only cells in focal planes at least 30 μm beneath the surface of the slice were monitored.

### Event selection and data analysis

Image analysis and automatic time-series event detection were accomplished with custom-written MATLAB (MathWorks, Natick, MA, USA) scripts. Regions of interest (ROIs) were manually selected from movies displaying the change in fluorescence of each pixel at each time point normalized by the mean fluorescence of the noise floor (ΔF/F_0_). The noise floor was defined for each pixel as all fluorescence intensity values within two standard deviations of the mean signal. All observable transfected cell somas were analyzed regardless of the presence of spontaneous events. Cell process ROIs were defined as larger than 1 μm^2^ with a maximum peak ΔF/F_0_ greater than double the standard deviation of the noise floor of the pooled data (2^*^σ = 45% ΔF/F_0_). Each ROI was modified to include only highly correlated pixels (*r* > 0.7). There were no overlapping ROIs. ΔF/F_0_ time-series plots were generated for each ROI by averaging ΔF/F_0_ values of each pixel at each time point. The event threshold was set to 2^*^σ. Events that occurred closely in time were treated as a single event. Events were merged if the minimum ΔF/F_0_ value between the event peaks was greater than half of the minimum peak ΔF/F_0_ of the two events. The full-width at half-maximum (FWHM) was calculated by measuring the width of the peak at half of the value of the peak ΔF/F_0_. FWHM is expressed in seconds (s).

### Statistics

Statistical analyses were performed with GraphPad Prism 6 (GraphPad Software, La Jolla, CA, USA). We used Student's *t*-test to compare means of normally distributed data, as determined by the Shapiro–Wilk normality test. Otherwise, the following non-parametric tests were used: the Kolmogorov–Smirnov test (KS-test) to compare cumulative distributions, the Mann–Whitney U test (MWU-test) to compare means; and the Kruskal-Wallis One-Way analysis of variance (KW-ANOVA) with Dunn's *post-hoc* test (Dunn's test) to compare medians between several groups. Statistics are presented as mean ± standard deviation unless otherwise noted. A significance level of *p* < 0.05 was used.

## Results

### A plasmid toolkit for recording calcium transients in the rat brain

Following *IUE*, transfected radial glia precursor cells in the subventricular wall of the lateral ventricle migrate, divide and differentiate into neurons, astrocytes, and oligodendrocytes throughout the cortex, hippocampus, and/or other brain regions (Tabata and Nakajima, 2001; Bai et al., 2003; Nakahira and Yuasa, 2005). Mammalian cells lack the ability to replicate plasmid DNA. Therefore, cells that continue to divide at a high rate throughout development, including astrocytes, eventually dilute the transfected plasmid and fail to express the delivered transgene. Transposon systems, such as *piggyBac*, have been exploited for effective transgene-integration into genomic DNA, resulting in high expression in astrocytes (Yoshida et al., 2010; Chen and LoTurco, 2012; Chen et al., 2014). In the presence of *piggyBac* transposase, single copies of ITR-flanked DNA are cut out of transfected plasmid DNA and inserted into random “TTAA” sites in diverse locations throughout genomic DNA (Fraser et al., 1995, 1996; Lorenzen et al., 2003) preferring transcriptionally active regions (Ding et al., 2005). Transgene-integration mediated by *piggyBac* has adopted the form of a paired plasmid co-transfection scheme consisting of a donor plasmid carrying an ITR-flanked transgene and a helper plasmid carrying *piggyBac* transposase which is not flanked by ITRs, and therefore does not integrate into genomic DNA (Lorenzen et al., 2003; Ding et al., 2005; Chen and LoTurco, 2012; Chen et al., 2014). A cell must receive both the donor plasmid and helper plasmid in order for the ITR-flanked transgene to integrate with high efficiency. We used *piggyBac* to stably express GCaMP (3, 6s, or 6f) throughout each transfected progenitor lineage including astrocyte and neuron populations.

GCaMP expression following *IUE* can be sparse and baseline fluorescence is dim due to low concentrations of unchelated calcium in resting cells. In order to facilitate detection of transfected cells at baseline activity levels, we incorporated tdTomato, a bright red fluorescent protein that localizes to cytosol and is readily detectable regardless of cell activity state. GCaMP and tdTomato were co-expressed in the same cells via IRES. We assembled a set of donor plasmids of the form ITR-CAG-GCaMP-IRES-tdTomato-ITR (Figure 1B; Table 1). Expression of *piggyBac* transposase was provided by the helper plasmid pPBase (Figure 1A; Wu et al., 2007).

**Table 1:** **A full toolset of plasmid variants for *IUE***.

**Plasmid name**	**ITR-flanked cassette**	**Subcellular target**	**Addgene #**
pPBC-LG6f	Lck-GCaMP6f	Plasma membrane	62808
pPBC-LG6s	Lck-GCaMP6s	Plasma membrane	62809
pPBC-LG6f-tdT	Lck-GCaMP6f-IRES-tdTomato	Plasma membrane	62807
pPBC-LG3-tdT	Lck-GCaMP3-IRES-tdTomato	Plasma membrane	62810
pPBC-G6f	GCaMP6f	Cytosol	62811
pPBC-G6s	GCaMP6s	Cytosol	62812
pPBC-G6f-tdT	GCaMP6f-IRES-tdTomato	Cytosol	62813
pPBC-G3-tdT	GCaMP3-IRES-tdTomato	Cytosol	62814

*All piggyBac ITR-flanked cassettes are driven by the CAG promoter. Plasmids are co-electroporated with a piggyBac transposase-expressing plasmid (Wu et al., 2007)*.

Standard GCaMP localizes to cytosol and may not be present in cellular processes where much of the synaptic activity-related calcium transients occur in astrocytes and neurons. The N-terminus of Lck, a lymphocyte-specific, membrane-spanning tyrosine kinase, has been shown to successfully target GCaMP2 and GCaMP3 to the plasma membrane and processes of astrocytes in culture and *in vivo* using viral transfection (Shigetomi et al., 2010a,b, 2013). We created a plasma-membrane targeted version of each plasmid (Figure 1B; Table 1). The N-terminus of GCaMP, and not tdTomato, was tagged with the N-terminus of Lck (Figure 1B). Therefore, most of the cytosolic compartment becomes filled with tdTomato and the borders of the cell can be readily detected. Each plasmid was independently co-electroporated with pPBase. All of the plasmid versions that we have constructed and submitted to Addgene are shown in Table 1.

### *In utero* electroporation robustly labels many brain regions

*IUE* can be used to express transgenes in astrocytes and neurons in various regions of the brain. Fluorescent proteins, such as GFP and RFP, have previously been expressed in the cortex and hippocampus of mice (Nakahira and Yuasa, 2005; Navarro-Quiroga et al., 2007; Yoshida et al., 2010; Pacary et al., 2012) and rats (Nakamura et al., 2006; Rosen et al., 2007) following *IUE*, allowing for lineage tracing of transfected cells. We electroporated pPBC-GFP and pPBase between gestational days 13.5–14.5 and used immunohistochemistry and confocal imaging to detect brain region and cell-type expression of GFP. It was sometimes difficult to identify electroporated astrocytes by overlap of the thin intermediate filament GFAP with cytosolic GFP. Therefore, in a subset of slices, we also labeled astrocyte-specific cytosol-localized (similar to GFP) Aldh1L1 to confirm astrocyte transfection (Lovatt et al., 2007; Cahoy et al., 2008; Yang et al., 2011). Electroporated neurons were identified by morphology. We successfully targeted pPBC-GFP to astrocytes and neurons throughout the brain including the hippocampus, dentate gyrus, nucleus accumbens, cortex, thalamus (Figures 1C–I), or striatum (not shown). Using the specific electroporation approach described in the Materials and Methods Section, the same patterns of astrocytic and neuronal transfection in the hippocampus and cortex could be reproduced between animals (Figure 1C). As expected, because they do not derive from lateral ventricle precursor cells, we did not observe any microglial transfection. We, likewise, electroporated the pPB-CAG-GCaMP3-IRES-tdTomato plasmid (Supplementary Movie 1). Transfected cells were readily identified with tdTomato (Figure 1H). Co-expression of GCaMP and tdTomato was confirmed in all transfected cells observed (Figure 1I). After optimizing the *IUE* technique for our laboratory, we can reliably electroporate the cortex and hippocampus of 80–100% of animals per litter.

### No overt immune response in the brain following *IUE*

Astrocytes respond to damage and immune activity by switching to a reactive state. This state, termed “reactive astrogliosis” is defined by increased GFAP expression, extended processes, and overlapping of astrocyte domains (Bushong et al., 2002; Oberheim et al., 2008). To test the possibility that *IUE* induces reactive astrogliosis, we compared GFAP expression in the cortex and hippocampus of pPBC-GFP-electroporated rats with unmanipulated age-matched controls (P18). We did not observe any signs of damage or reactive astrogliosis in either group. Astrocytes in both groups appeared normal, only had a few thin GFAP positive processes and did not overlap with neighboring astrocyte domains (Bushong et al., 2002). In addition, it is important to note that astrocytes located near transfected cells did not display a reactive morphology (Figures 2A–F).

**Figure 3:**
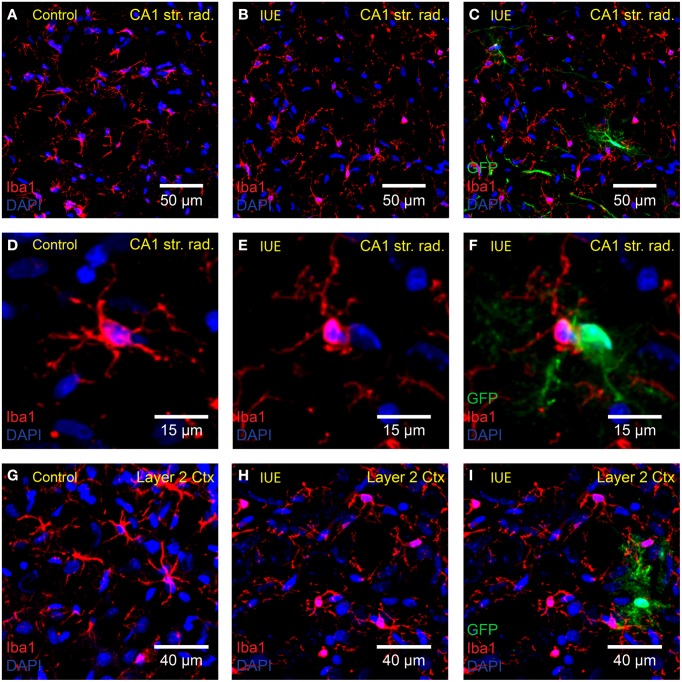
**Microglia are not transfected or overtly activated following *IUE***. Confocal imaging of anti-Iba1 (red) stained microglia in the **(A–F)** hippocampal CA1 stratum radiatum and **(G–I)** cortex layer 2 of **(A,D,G)** unelectroporated brains and **(B,C,E,F,H,I)** following *IUE* of CAG-GFP-IRES-Neo. The right-hand column displays images in the middle column overlaid with the green channel (GFP-transfected cells). **(C,F,I)** As expected, anti-Iba1 did not overlap with GFP. Iba1-positive cells near GFP-transfected cells did not appear to be activated. DAPI staining is shown in blue.

In the healthy brain, resting microglia exhibit a “ramified” morphology defined by long thin dynamic processes that scan the local area around the stationary soma (Davalos et al., 2005; Nimmerjahn et al., 2005). Following activation, microglia convert to a reactive state, delineated by retraction of processes, and adoption of an “amoeboid” shape. We did not detect overt signs of microglial activation with Iba1 staining (Figures 3A–I) of cortical and hippocampal tissue obtained from GFP-transfected P18 rats (*n* = 4 *IUE* animals and *n* = 3 controls). Microglia in the *IUE* brain and age-matched controls retained a resting ramified morphology suggesting that there was no major neuroimmune response to the *IUE* procedure.

### Robust spontaneous calcium activity in organotypic slice cultures

Hippocampal OSCs provide an *in vitro* preparation which can be used for chronic studies of intact neural circuits (Khalilov et al., 1997), albeit in the relatively immature brain. OSCs have been used as an *in vitro* model of epileptogenesis (Dyhrfjeld-Johnsen et al., 2010). Following slicing-induced deafferentiation, neuronal networks rearrange, and exhibit spontaneous epileptiform-like activity which can be visualized with calcium indicators (Sabolek et al., 2012). In order to test our plasmids in this system, we prepared OSCs from electroporated P10 rats and imaged astrocytic and neuronal calcium activity with Lck-GCaMP6f (pPBC-LG6f plasmid) 7–10 days later (*n* = 3 slice cultures).

**Figure 4:**
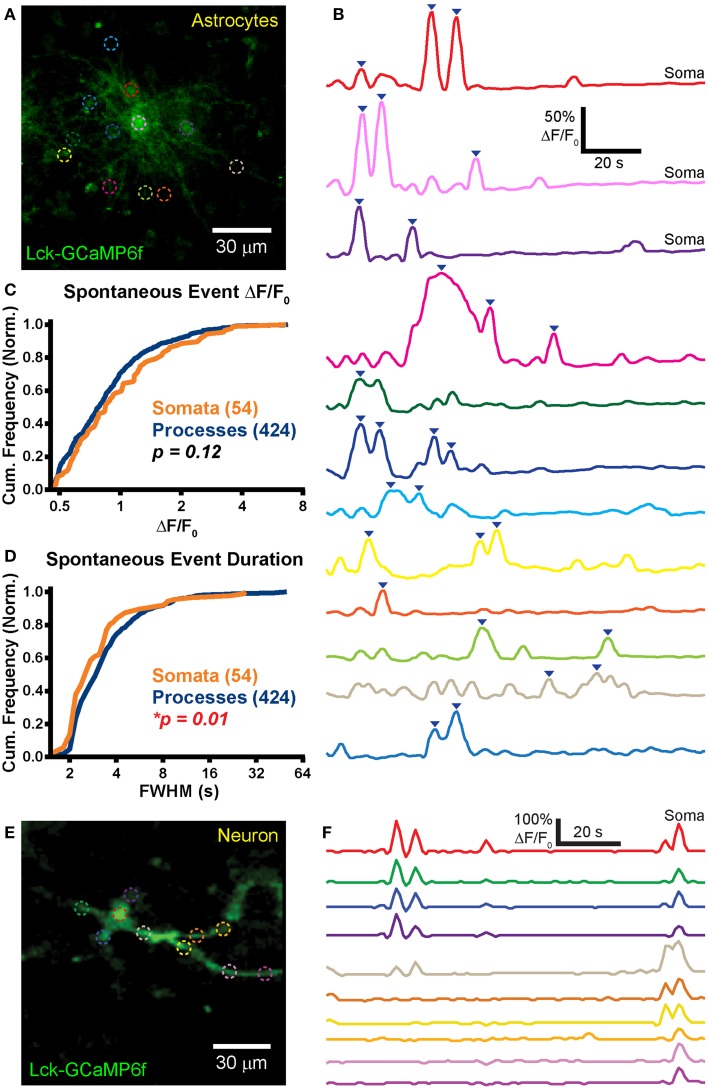
**Robust spontaneous transients are observed in astrocytic somas and processes in hippocampal organotypic slice cultures. (A)** Example mean calcium activity projection image of astrocytes expressing Lck-GCaMP6f in a hippocampal organotypic slice culture. ROIs are denoted by dashed circles including three adjacent somas. **(B)** ΔF/F_0_ traces of ROIs shown in **(A)**. Line colors in **(B)** correspond to ROI colors in **(A)**. Inverted blue arrows mark peaks that reached threshold and were included as events for analysis. “Merged” events, which encompass two proximate peaks, were counted as a single long event (see dark pink and dark green ROIs). **(C)** A cumulative histogram displaying the relative frequency (event counts normalized by total events in each structure) of spontaneous event peak ΔF/F_0_ values. The number of total events in each structure is shown in parentheses next to the structure name. **(D)** A cumulative histogram displaying spontaneous event duration. Soma data is shown in orange and process data in blue. The distribution of each measurement was compared between somas and processes. The calculated *p-value* from a Kolmogorov–Smirnov test is shown in the plot. Red text denotes significance and black text denotes no significance detected at a level of *p* < 0.05. **(E)** Example mean calcium activity projection of a neuron expressing Lck-GCaMP6f. **(F)** ΔF/F_0_ traces corresponding to ROIs shown in **(E)**.

Unlike the normal healthy morphology that was observed in GFP-transfected astrocytes in slices obtained from the mature rat brain (Figures 1, 2), OSC astrocytes exhibited a reactive morphology and organization (Figure 4A; Supplementary Movie 2). We observed significant astrocytic domain overlap between adjacent astrocytes to the point where it was difficult to assign processes to a specific cell. The example in Figure 4A shows three putative astrocytes sharing the same domain. Astrocytic processes were also noticeably extended at these time points (Supplementary Movie 2). Morphology indicative of astrocyte reactivity in the hippocampus of OSCs was apparent.

Frequent spontaneous calcium activity was observed in both astrocytic somas (*f* = 1.9 ± 1.5 events/ROI/min; *n* = 54 events in 20 somas; *f*, event frequency; ROI, region of interest) and processes (*f* = 1.2 ± 1.3 events/ROI/min; *n* = 424 events in 168 ROIs). In stark contrast to our previously reported acute brain slice (ABS) data obtained from the GFAP-CreER; PC::G5-tdT mouse (Gee et al., 2014), spontaneous OSC somatic events were significantly more frequent than process events (*p* = 0.002; Student's *t*-test). In fact, we detected above-threshold calcium activity in all 20 OSC astrocytic somas selected for imaging [mean duration of an imaging session (*T*_mean_) = 114 s/cell].

Large spontaneous astrocyte event peak ΔF/F_0_ values were measured for both somas (ΔF/F_0_ = 117 ± 102%) and processes (ΔF/F_0_ = 100 ± 73%). The distributions of OSC event peak ΔF/F_0_ did not differ significantly between the cellular compartments (Figure 4C; *p* = 0.12, *D* = 0.14, KS-test) nor did the means (*p* = 0.26, MWU-test). Event duration was also measured for all spontaneous events in somas (FWHM = 3.2 ± 2 s) and in processes (FWHM = 4.3 ± 4.8 s). The event duration distribution of somas was left-shifted relative to that of processes (Figure 4D; *p* = 0.01, *D* = 0.23, KS-test) and the mean event duration was larger in processes than in somas (*p* = 0.001, MWU-test).

Spontaneous events were also observed in OSC neurons (Figure 4E; *n* = 5). Activity in different ROIs within a single cell appeared to be highly correlated (Figure 4F), reflecting action potential-induced intracellular calcium signaling. We detected no difference in spontaneous neuronal soma (*f* = 1.4 ± 0.6 events/soma/min; *n* = 5 somas) and process (*f* = 2 ± 2.1 events/ROI/min; *n* = 16 ROIs) event frequency (*p* = 0.53, Student's *t*-test).

### Frequent spontaneous events in cortical astrocytic processes of young animals

**Figure 5:**
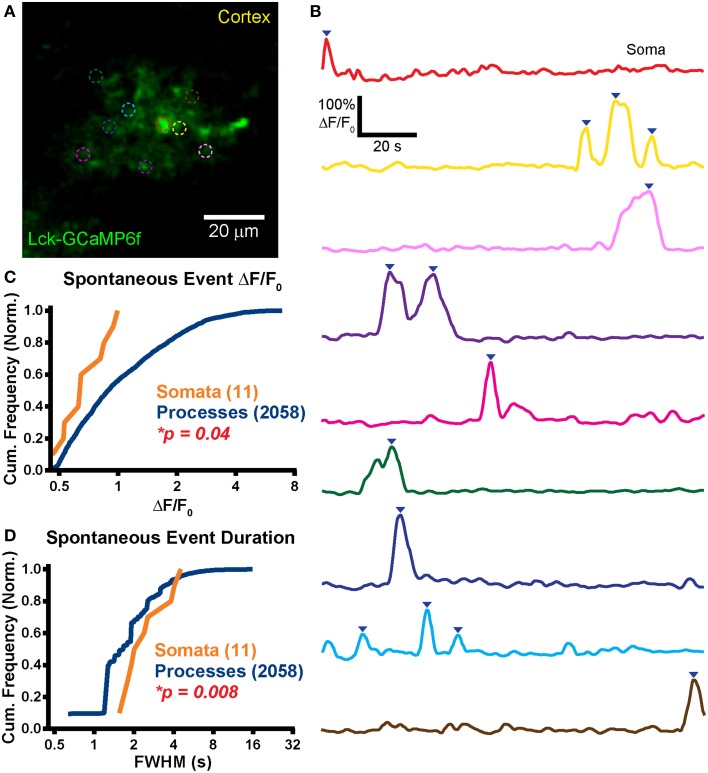
**Large spontaneous events in astrocytic processes but not somas. (A)** Example mean calcium activity projection image of a cortical astrocyte expressing Lck-GCaMP6f in an acute brain slice obtained from a P34 rat. As in Figure 4, ROIs are denoted by dashed circles. **(B)** ΔF/F_0_ traces of ROIs shown in **(A)**. Line colors in **(B)** correspond to dashed circle colors in **(A)**. Inverted blue arrows mark peaks that reached threshold and were included as events for analysis. “Merged” events were counted as a single long event (see green ROI). **(C)** A cumulative histogram displaying the relative frequency (event counts normalized by total events in each structure) of spontaneous event peak ΔF/F_0_ values. The total events in each structure is shown in parentheses next to the structure name. **(D)** A cumulative histogram displaying spontaneous event duration. Soma data is shown in orange and process data in blue. The distribution of each measurement was compared between somas and processes. The calculated *p-value* from a Kolmogorov–Smirnov test is shown in each plot. Red text denotes significance and black text denotes no significance detected at a level of *p* < 0.05.

ABS preparations are relatively quiet with regard to spontaneous neuronal activity. This is likely due to the acute removal of synaptic input to neurons within the slice. Although spontaneous neuronal activity is infrequent in ABS, astrocytes display frequent spontaneous calcium events. We, therefore, employed the pPBC-LG6f plasmid for detecting spontaneous cortical astrocytic activity in ABS (*n* = 6 slices) obtained from young mature rat brains (P34-P70; “young” group). ABS astrocytes did not exhibit the reactive characteristics observed in OSCs. Cortical astrocytic processes were relatively short and did not overlap with adjacent astrocytic processes. It was usually straightforward to correctly assign a process to the correct soma in ABS (Figure 5A; Supplementary Movie 3).

In line with our previous reported data collected in mouse ABS (Gee et al., 2014) and in contrast to the OSC data reported above, we observed very few spontaneous calcium events in astrocytic somas (*f* = 0.8 ± 1.2 events/soma/min; *n* = 11 events in 9 somas). Only 4 of 9 imaged somas displayed spontaneous events (*T_mean_* = 123 s). The pooled data soma event frequency, which takes into account the imaging duration of soma ROIs with no events, was 0.5 events/soma/min. Spontaneous process event frequency (Figure 5B; *f* = 3 ± 1.8 events/ROI/min, *n* = 2058 events in 287 ROIs) was significantly higher than in somas (*p* < 0.0001; Student's *t*-test).

The infrequent somatic events that we did detect tended to be small (ΔF/F_0_ = 71 ± 18%) whereas process events appeared much larger (ΔF/F_0_ = 120 ± 88%). The ΔF/F_0_ distribution of somatic events was left shifted relative to process events (Figure 5C; *p* = 0.04, *D* = 0.42, KS-test) but the difference in means failed to reach significance (*p* = 0.07, MWU-test).

Somatic (FWHM = 2.6 ± 1.1 s) and process event duration (FWHM = 2 ± 1.3 s) were also measured. The event duration distribution of somas was right-shifted relative to that of processes (Figure 5D; *p* = 0.01, *D* = 0.50, KS-test) and mean somatic event duration was longer than in processes (*p* = 0.01, MWU-test). We did not observe supra-threshold spontaneous activity in neurons.

### Stable and functional expression of GCaMP6f in the adult rat cortex

For technical reasons, most published calcium imaging data have been collected from perinatal or adolescent animals. Because of the vast differences in cellular function, connectivity and gene expression between the young and adult brain (Sun et al., 2013), it is important to develop methods for recording activity in tissue obtained from animals of a wide range of ages (Yuste and Katz, 1991; Schwartz et al., 1998; Peterlin et al., 2000; Reeves et al., 2011). To test GCaMP6f function and expression stability in the rat brain following *IUE*, we prepared ABS from older animals (P125-P127; “old” group) electroporated with the pPBC-LG6f plasmid during gestation.

**Figure 6:**
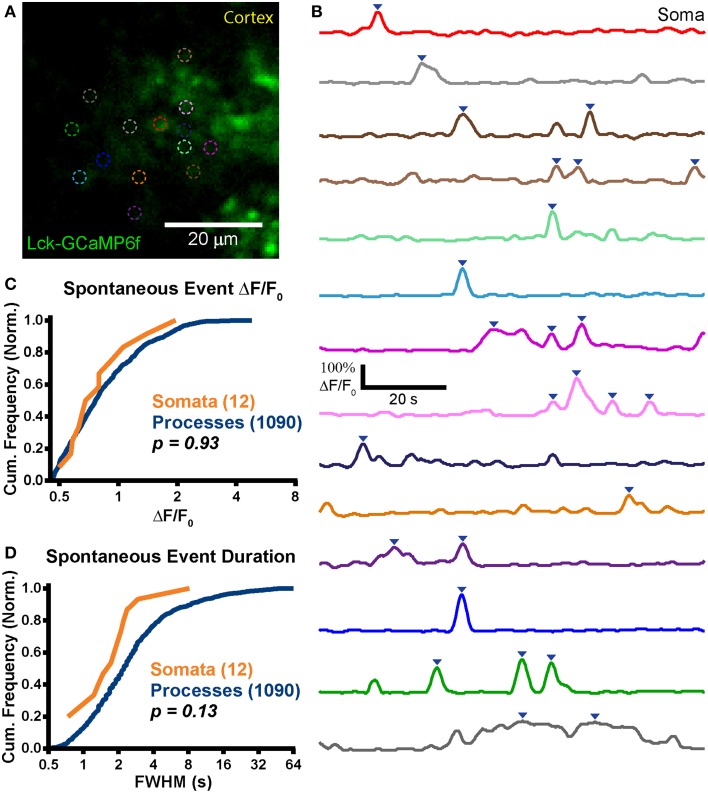
**Stable expression in adult animals. (A)** Example mean calcium activity projection image of a cortical astrocyte expressing Lck-GCaMP6f in an acute brain slice obtained from a P125 rat. As in Figures 4, 5, ROIs are denoted by dashed circles. **(B)** ΔF/F_0_ traces of ROIs shown in **(A)**. Line colors in **(B)** correspond to dashed circle colors in **(A)**. Inverted blue arrows mark peaks that reached threshold and were included as events for analysis. “Merged” events were counted as a single long event (see dark gray ROI). **(C)** A cumulative histogram displaying the relative frequency (event counts normalized by total events in each structure) of spontaneous event peak ΔF/F_0_ values. The total events in each structure is shown in parentheses next to the structure name. **(D)** A cumulative histogram displaying spontaneous event duration. Soma data is shown in orange and process data in blue. The distribution of each measurement was compared between somas and processes. The calculated *p-value* from a Kolmogorov–Smirnov test is shown in the plot. Red text denotes significance and black text denotes no significance detected at a level of *p* < 0.05.

We observed expression and strong Lck-GCaMP6f signal in the adult rat brain (*n* = 2 animals; Figures 6A,B). Similar to ABS obtained from younger animals, spontaneous somatic calcium events in astrocytes were infrequent in ABS prepared from older animals (*f* = 0.8 ± 3.3 events/soma/min; *n* = 12 events in 17 somas; *T_mean_* = 141 s/cell) but significantly higher in processes (*f* = 2.5 ± 2.8 events/ROI/min; *n* = 1090 events in 293 ROIs; *p* = 0.04, Student's *t*-test). Interestingly, only 2 of 17 imaged somas displayed activity above the ΔF/F_0_ threshold and 11 of the 12 total events were observed in a single soma. The peak ΔF/F_0_ values were 88 ± 42% in somas and 94 ± 52% in processes. We detected no difference between the peak ΔF/F_0_ distributions (Figure 6C; *p* = 0.92, *D* = 0.16, KS-test) or means (*p* = 0.90, MWU-test) in somas and processes. We also did not detect any statistical differences between the distributions (Figure 6D; *p* = 0.13, *D* = 0.34, KS-test) or means of event FWHM (*p* = 0.06, MWU-test) in somas (FWHM = 2.1 ± 2 s/event) and processes (FWHM = 4.1 ± 6.2 s/event). Similar to the younger ABS group, we also did not observe supra-threshold spontaneous activity in neurons.

### Differences in astrocyte spontaneous event frequency across preparations

**Figure 7:**
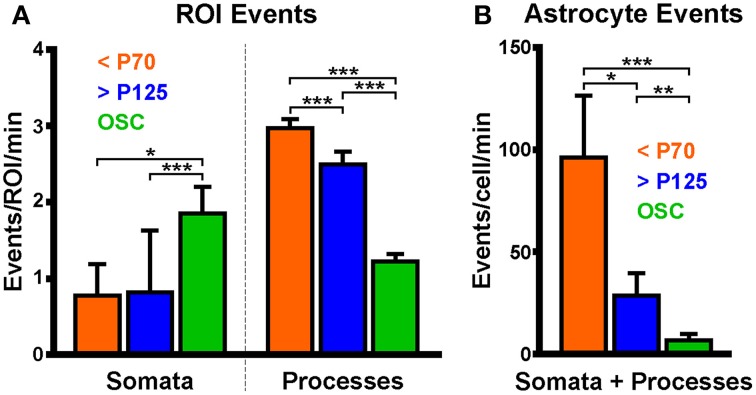
**Astrocytic process event frequency varies between preparations. (A)** Spontaneous astrocyte event frequency per ROI in somas and processes. Data were separated into three groups according to which preparation or age of rat was used. **(B)** Spontaneous astrocyte event frequency per cell. Colored bars correspond to data collected from acute brain slices obtained from young (orange; <P70) or old (blue, >P125) animals or from hippocampal organotypic slice cultures (green; OSC). Plots display mean ± SEM. Kruskal-Wallis ANOVA using Dunn's *post-hoc* test was used to detect differences in the medians of each group. Significant differences between groups are denoted by ^*^p < 0.05, ^**^p < 0.01, and ^***^p < 0.0001.

The spontaneous astrocyte event rate for each ROI (events/ROI/min) in the three groups (young, old and OSC) was compared. A significant difference in the median somatic event rate was detected between groups (reported as median (minimum: maximum) events/soma/min; young: 0 (0: 3.7), *n* = 11 events in 9 somas; old: 0 (0: 13.6), *n* = 12 events in 17 somas; OSC: 1.19 (0.5: 5.2), *n* = 54 events in 20 somas; *p* < 0.0001, KW-ANOVA). OSC somas exhibited a higher spontaneous event frequency than either of the two ABS groups (Figure 7A, left; OSC > young, *p* = 0.03; OSC > old, *p* < 0.0001; Dunn's post-test) but no difference was detected between the two ABS groups (young vs. old, *p* = 0.55; Dunn's post-test). Differences in the median process event rate were detected between the groups [young: 2.8 (0.1: 12.1) events/ROI/min, *n* = 2058 events in 287 ROIs; old: 1.5 (0.3: 18.5), *n* = 1090 events in 288 ROIs; OSC: 0.6 (0.2: 7.2), *n* = 424 events in 167 ROIs; *p* < 0.0001, KW-ANOVA]. Inter-group comparisons revealed significant differences in the medians between all groups (Figure 7A, right; young > OSC, *p* < 0.0001; old > OSC, *p* < 0.0001; young > old, *p* < 0.0001; Dunn's post-test).

The spontaneous event rate per cell in each group was also compared. All cells which could be distinguished as independent cells were included whether or not we could visualize the soma. A significant difference was detected in the whole astrocyte (soma and processes) event rate between groups [Figure 7B; young: 67.4 (9.6: 303.3) events/cell/min, *n* = 2069 events in 9 cells; old: 14.6 (0.8: 260.5), *n* = 1102 events in 26 cells; OSC: 3.1 (0.5: 57), *n* = 478 events in 45 cells; *p* < 0.0001, KW-ANOVA]. The young group astrocytes displayed much higher spontaneous event frequency than either the old or OSC groups while the OSC group displayed lower spontaneous event frequency than the ABS groups (young > OSC, *p* < 0.0001; old > OSC, *p* = 0.005; young vs. old, *p* = 0.03; Dunn's post-test).

## Discussion

We have demonstrated the suitability of our plasmid toolset for stable and functional expression of GECIs in the rat brain at various ages, in different brain regions, in different cell types, and in different preparations for imaging without the use of viral vectors. Our toolset of novel genetic constructs provides flexibility for the user, who can choose among three different versions of GCaMP (3, 6s, or 6f), with or without tdTomato co-expression and targeting to either the cytosol or plasma membrane (Table 1). *IUE* has previously been described as a method of tracking migration of neuron progenitors from the lateral ventricles to cortex (Bai et al., 2003) or to hippocampus (Navarro-Quiroga et al., 2007) and has also been used for GECI delivery (Tian et al., 2009). Transposon systems have previously been combined with *IUE* to stably express transgenes in astrocytes, as well as neurons, until at least P27 (Yoshida et al., 2010; Chen and LoTurco, 2012). We have, on the other hand, demonstrated that the combination of *IUE* with *piggyBac* results in stable astrocytic transgene expression well into adulthood (at least P127), comparable to what has been reported in neurons (Navarro-Quiroga et al., 2007). Our toolset adds to the existing available tools via inclusion of updated versions of GCaMP and the option of the fluorescent protein, tdTomato. In order to characterize the plasmids and to provide an example of their utility, we measured astrocytic and neuronal spontaneous calcium activity in two different preparations including in brain slices prepared from either young or old adult animals.

The role of spontaneous astrocytic activity is not well-understood but likely reflects changes in the local environment with respect to pH, temperature, oxygenation, immunological processes, and neuronal activity in addition to many other parameters (Wang et al., 2006a,b; Schipke et al., 2008; Haustein et al., 2014). Our results, while only preliminary, are nonetheless intriguing. Interestingly, we observed frequent somatic activity in OSCs and only rare events in both ABS groups. Similarly, we observed supra-threshold neuronal events in OSCs and not in ABS, indicating a possible relationship between spontaneous astrocytic somatic events and local neuronal-network activity. In contrast, astrocytic process events were much more frequent than somatic events in the ABS groups in which we did not observe significant neuronal activity, suggesting that process events may be relatively neuronal-activity independent. Qualitatively, OSC events encompassed a larger cellular volume than events in ABS, which were temporally shorter. Activity within and between cells in OSCs appeared to be more highly correlated than in ABS, possibly reflecting oscillating activity of OSC neuronal networks (Supplementary Movie 2). These results will need to be explored in more depth in future studies of spontaneous calcium activity in astrocytic-neuronal networks. However, they demonstrate the types of information which can be gleaned from combining our plasmids with *IUE* for imaging activity in the rat brain.

*IUE* is a practical and flexible method for delivering GECIs to the rat brain. By altering any of several parameters, such as timing of the *IUE* procedure with respect to embryonic development and spatial paddle placement with respect to the developing brain, different regions and cell types can be targeted (LoTurco et al., 2009; Yoshida et al., 2010). Once mastered, *IUE* can be performed expeditiously and provide a continuous supply of rats, in a timely manner, expressing GECIs or other genetically encoded tools for experiments in specific regions of the brain. In our hands, the cortex and hippocampus can be reliably targeted in 80–100% of animals in each litter.

Delivery of GECIs via *IUE* exhibits several advantages over viral-based methods. First, and perhaps most importantly, the plasmids we describe here are too large to be delivered via adeno-associated viruses. For comparison, it has been demonstrated that *piggyBac* can transpose elements up to 100 kb (Li et al., 2011b). Second, although both *IUE* and viral-based methods require invasive injections, the timing of these injections is likely important with regard to consequential inflammation and tissue damage. *IUE* injections are performed when the lateral ventricles are located superficially in the brain. Thus, only a minimal amount of tissue is disturbed by the injection. Third, the embryonic brain is much more plastic than the mature brain and so healing potential is presumably more favorable with *IUE*. Therefore, *IUE* injections likely cause less tissue damage than post-natal AAV injections. Fourth, whereas expression levels via *IUE* remain stable for months, transfection with viruses can lead to expression that is so high as to be toxic (Howard et al., 2008). Fifth, cloning for *IUE* is faster than for viruses and involves minimal biosafety concerns. Finally, in experiments of animal models of neurological disorders such as epilepsy, wherein inflammation is a critical component of the pathophysiology, repeated injections into adult tissue of viral vectors may confound interpretations, as the very act of physical injections can induce local inflammatory responses. Thus, performing *IUE* weeks to months prior to the experiments in question may be advantageous, especially as work presented here has demonstrated that there are no overt signs of inflammation in or near transfected cells.

However, there are also several drawbacks to the *IUE* technique. First, targeting and expression of plasmids is very sensitive to the injected plasmid concentration, timing of electroporation, and the position of paddles with respect to the brain (Navarro-Quiroga et al., 2007; Yoshida et al., 2010). Second, while astrocytes and neurons are readily transfected via this approach, *IUE* of lateral ventricular precursors will not transfect microglia, which are derived from hematopoietic stem cells. Third, it is difficult to confirm successful transfection following *IUE* which emphasizes the importance of empirically determining precise plasmid concentration, timing of *IUE* and paddle placement in order to replicate expression patterns.

The ability to target GECIs to genetically defined cells and subcellular compartments grants the *IUE* technique an advantage over bulk loading of synthetic dyes into brain tissue. Cre-lox technology has been integrated into plasmid systems and cell-type specific promoters have been used to drive transgene expression (Matsuda and Cepko, 2007). As has been demonstrated with Lck, GECIs can be targeted to specific subcellular compartments for recording of calcium activity in precisely defined regions of a cell (Shigetomi et al., 2010a). This allows for measurement of many different types of calcium activity which may not be accessible with synthetic dyes. Other N-terminal tags already exist that could conceivably be incorporated into these constructs as well (Li et al., 2011a).

Because integration of new reporters of cellular activity following *IUE* is only limited by the time required for cloning plasmid DNA, many reporter plasmids can be generated and expressed expeditiously. Other types of genetically encoded indicators, such as red-shifted calcium indicators, sypHTomato, and voltage sensitive fluorophores could readily be incorporated to expand the *IUE* toolset (Kralj et al., 2012; Li and Tsien, 2012; Wu et al., 2013). However, this toolset, as described, should contribute to the advancement of our understanding of astrocytic and neuronal activity at many spatial scales in the young or adult rat brain.

## Author contributions

JG, PT, KW, and JW planned and oversaw the project; JG and PT designed the plasmids; JG, SM, SP, and CC built the plasmids; MG and KF performed surgeries; MT, JG, and KF performed immunohistochemistry and confocal imaging; JG, MG, RS, and ME performed two-photon imaging; JG performed data analysis and wrote the manuscript with editing and additional writing from JW, KW, PT, SP, MT, and MG; JW, KW, PT, and MC provided resources; All authors proofread the manuscript.

### Conflict of interest statement

The authors declare that the research was conducted in the absence of any commercial or financial relationships that could be construed as a potential conflict of interest.

## Supplementary material

Supplementary Movie 1 **Spontaneous activity in an astrocyte expressing Lck-GCaMP3 and tdTomato in an acute brain slice**. Frequent localized spontaneous calcium events can be observed in both the thick proximal and small distal processes. This image was captured usinga 60× objective.

Supplementary Movie 2 **Spontaneous calcium activity in hippocampal astrocytes expressing Lck-GCaMP6f in an organotypic slice culture**. OSCs were prepared from a P10 animal and imaged 1 week later. Calcium activity within and between astrocytes appeared to be highly correlated. Astrocytic domains were highly overlapped and astrocytic processes were extended, suggesting that these organotypic slice culture astrocytes were reactive. In this movie, several putative neuronal events can be observed. The highly active astrocytes in the center of the field are analyzed further in Figures 4A,B.

Supplementary Movie 3 **Spontaneous activity in a cortical astrocyte expressing Lck-GCaMP6f in an acute brain slice**. Slices were obtained from a P34 animal. Astrocyte calcium events appeared more random and much smaller than those seen in organotypic slice cultures. Localized events with large fluorescence changes were frequently observed throughout astrocyte processes with only sparse somatic activity. We did not observe astrocytes with overlapping domains or extended processes, consistent with a non-reactive phenotype. Neuronal events (not shown in this movie) were very sparse with and small in amplitude. The upper right astrocyte in this movie is used as the example cell in Figures 5A,B.

## Abbreviations

ABS: acute brain slice
Aldh1L1: aldehyde dehydrogenase 1 family, member L1
CAG: cytomegalovirus early enhancer/chicken beta actin
FWHM: full-width at half-maximum
GFAP: glial fibrillary acidic protein
GFP: green fluorescent protein
IRES: internal ribosomal entry site
ITR: inverted terminal repeat
*IUE*: *in utero* electroporation
KS: Kolmogorov–Smirnov
KW-ANOVA: Kruskal-Wallis One-Way analysis of variance
Lck: lymphocyte-specific protein tyrosine kinase
MWU: Mann–Whitney U
OSC: organotypic slice culture
PB: *piggyBac*
ROI: region of interest.
